# Generation of Spheres from Dental Epithelial Stem Cells

**DOI:** 10.3389/fphys.2017.00007

**Published:** 2017-01-19

**Authors:** Despoina Natsiou, Zoraide Granchi, Thimios A. Mitsiadis, Lucia Jimenez-Rojo

**Affiliations:** ^1^Orofacial Development and Regeneration, Centre for Dental Medicine, Institute of Oral Biology, University of ZurichZurich, Switzerland; ^2^Genomescan B.V.Leiden, Netherlands

**Keywords:** incisor, tooth, regeneration, epithelial stem cells, mouse, spheres, stem cell niche, 3D culture

## Abstract

The *in vitro* three-dimensional sphere model has already been established as an important tool in fundamental sciences. This model facilitates the study of a variety of biological processes including stem cell/niche functions and tissue responses to injury and drugs. Here we describe the complete protocol for the *in vitro* formation of spheres originated from the epithelium of rodent incisors. In addition, we show that in these spheres cell proliferation is maintained, as well as the expression of several key molecules characterizing stem cells such as Sox2 and p63. These epithelial dentospheres could be used as an *in vitro* model system for stem cell research purposes.

## Introduction

Adult stem cells reside in specific and well-defined areas of most organs and tissues called stem cell niches. Niches provide to stem cells proper signals in order to regulate their function and maintenance according to the requirements of each specific tissue (Li and Xie, 2005; Pagella et al., 2015; Kirkeby et al., 2016). Therefore, the niches contain sources of undifferentiated cells that are involved in both tissue homeostasis and reparative processes after injury (Jiménez-Rojo et al., 2012; Van Keymeulen and Blanpain, 2012). The presence of stem cells is more important in organs with rapid renewal such as hairs, skin and intestine (Blanpain and Fuchs, 2014; Tetteh et al., 2015). The identification of different epithelial stem cell populations with various functions and plasticity during homeostasis and regeneration suggests the complexity of adult epithelial stem cell niches (Blanpain and Fuchs, 2014; Tetteh et al., 2015).

Although monolayer stem cell cultures were established more than three decades ago, three dimensional (3D) *in vitro* systems have recently emerged in order to preserve the physiological microenvironment of the cultured stem cells, thus providing an important tool for both basic and clinical research (Edmondson et al., 2014; Fatehullah et al., 2016; Kretzschmar and Clevers, 2016). Sphere-forming assays have been used to define stemness of adult epithelial cells within organs and tissues (e.g., intestine, mammary glands, and lungs; Dontu et al., 2003). In contrast to the two-dimensional (2D) monolayer culture, 3D culture systems allow cells to grow by forming aggregates/spheroids or organoids (Sasai et al., 2012; Edmondson et al., 2014).

In rodent incisors, dental epithelial stem cells reside in their most posterior part, the cervical loop area (Mitsiadis et al., 2007). The niche formed at the cervical loop is composed by a variety of epithelial cell populations and is separated from the surrounding mesenchyme by a basement membrane (Kieffer-Combeau et al., 2001; Mitsiadis et al., 2007). Hereby, we describe a technique to obtain epithelial dentospheres from the cervical loop of the continuously growing mouse incisor. This method allows evaluating the stemness and plasticity of dental epithelial cells, thus providing with essential information before proceeding with cell-based regenerative approaches in clinics.

## Materials and methods

All mice (C57Bl/6) were maintained and handled according to the Swiss Animal Welfare Law and the study was approved by the Cantonal Veterinary office, Zurich (License 11/2014).

### Dissection of cervical loops from mouse incisors (stereomicroscope)

- Sacrifice mice by decapitation.
- Separate the maxilla and the mandible with a single-edge razor blade.
- Separate the two hemimandibles.
- Remove the soft tissues around the mandibular alveolar bone.
- Remove the bone until the incisor is totally exposed.
- Press with dissection forceps the bone beneath the cervical loop region to avoid that it remains attached during the next step.
- Take the apical part of the incisor with the forceps and pull out the incisor.
- Incubate the incisors in Dispase (2 mg/ml) and DNase (20 U/ml) solution in Hank's Balanced Salt Solution (HBSS) for 20 min at RT.
- Dissect the posterior area of the incisor that contains the cervical loop area with the help of needles (25G) by separating it mechanically from the mesenchyme.
- Cut the cervical loop area and add it to a 15 ml falcon tube containing 1 ml of 10% Calf Serum (CS) in Phosphate Buffered Saline (PBS).

### Isolation of single dental epithelial cells (under the laminar flow)

xi. Centrifuge the cervical loops in 10% CS/PBS at 300 g for 5 min at RT.
xii. Wash with PBS and centrifuge at 300 g for 5 min at RT.
xiii. Add 250 μl of 0.25% Trypsin (in Leibovitz's L-15 (L-15) Medium) and incubate 20 min at 37°C.
xiv. Pipet up and down.
xv. Add 500 μl DNase I (2 U/ml in L15 medium) and incubate 5 min at 37°C.
xvi. Add 10% CS/PBS.
xvii. Centrifuge at 300 g for 5 min.
xviii. Resuspend the cells in PBS and filter them through a 40 μm cell strainer.
xix. Count the cells.

### Embedding of single dental epithelial stem cells in matrigel (under the laminar flow)

xx. An aliquot of BD (BD Biosciences) Matrigel™ Basement Membrane Matrix (referred to as Matrigel) should be transferred from −20° to 4°C the afternoon before the day of the experiment.
xxi. Centrifuge the cells at 300 g for 5 min.
xxii. Remove the supernatant and resuspend the cells in the desired volume of culture medium:
  – Dulbecco's Modified Eagle's Medium/Nutrient F-12 Ham (DMEM/F12) medium (no phenol red) with B-27 Supplement (1X), Epidermal Growth Factor (EGF) (20 ng/ml), basic Fibroblast Growth Factor (bFGF) (20 ng/ml) and 1% Penicillin/Streptomycin or
  – Keratinocyte serum free medium (KSFM) supplemented with EGF and Bovine Pituitary Extract (BPE) and 1% Penicillin/Streptomycin. For each well (12 well/plate), resuspend 20,000 cells in 50 μl of sphere-forming medium.
xxiii. Add 50 μl of Matrigel to the 50 μl of cells/medium (keeping the tube on ice). Mix gently.
xxiv. Add the 100 μl of Matrigel/cells/medium in the middle of a well (in a 12 well/plate).
xxv. Place the 12 well/plate 1 h at 37°C (in the incubator) to allow the Matrigel to solidify.
xxvi. Add 1 ml of sphere-forming medium to each well.
xxvii. Add 500 μl of fresh medium every second/third day.

### Harvesting the resulting spheres and processing them for histology

xxviii. Spheres can be harvested 7–10 days after plating the cells.
xxix. Add 500 μl of Dispase II (1 mg/ml) to each well and place the plate 30 mins at 37°C (in the incubator).
xxx. Transfer the volume contained in one well to a 15 ml falcon tube.
xxxi. Centrifuge at 800 g for 5 min.
xxxii. Remove the supernatant and resuspend in 1 ml of 4% PFA. Incubate 20 mins at room temperature.
xxxiii. Centrifuge at 800 g for 5 min.
xxxiv. Remove the supernatant and resuspend in 1 ml of PBS.
xxxv. Centrifuge at 800 g for 5 min.
xxxvi. Remove the supernatant and add the pellet into a plastic cryomold.
xxxvii. Add agarose 1% in the cryomold and let it solidify at room temperature.
xxxviii. Remove the agarose block containing the spheres, place it inside a cassette and process it for paraffin embedding.
xxxix. Section resulting paraffin blocks at 5 μm and perform hematoxylin-eosin staining.
xl. Analyse the slides with Leica DM6000 FS microscope and take pictures with the Leica DFC420C camera.

## Results

**Figure 1:**
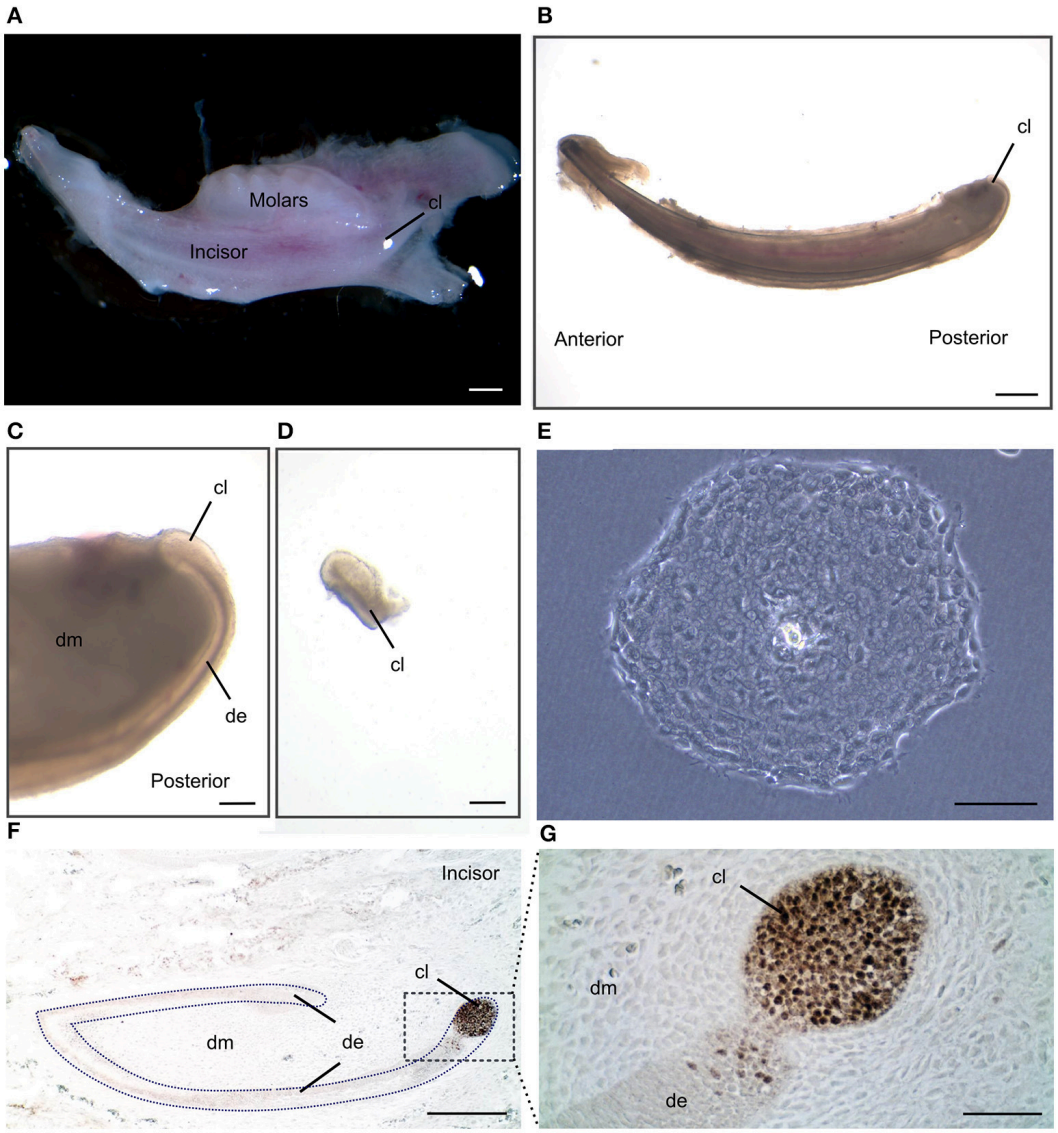
**Isolation of dental epithelial stem cells from mouse incisor cervical loop. (A)** Mouse mandibular alveolar bone containing incisor and molars. **(B)** Isolated postanal mouse incisor. **(C)** Detail of the posterior part of the incisor where the cervical loop is located. **(D)** Isolated cervical loop. **(E)** Colony derived from a single dental epithelial stem cell from mouse cervical loop cultured in a 2D culture system. **(F)** Immunohistochemistry against the dental epithelial stem cell marker Sox2 and **(G)** a higher magnification of the cervical loop area. Scale bars: 500 μm in **(A,B)**; 100 μm in **(C,D)**; 200 μm in **(E,F)**; 20 μm in **(G)**.

**Figure 2:**
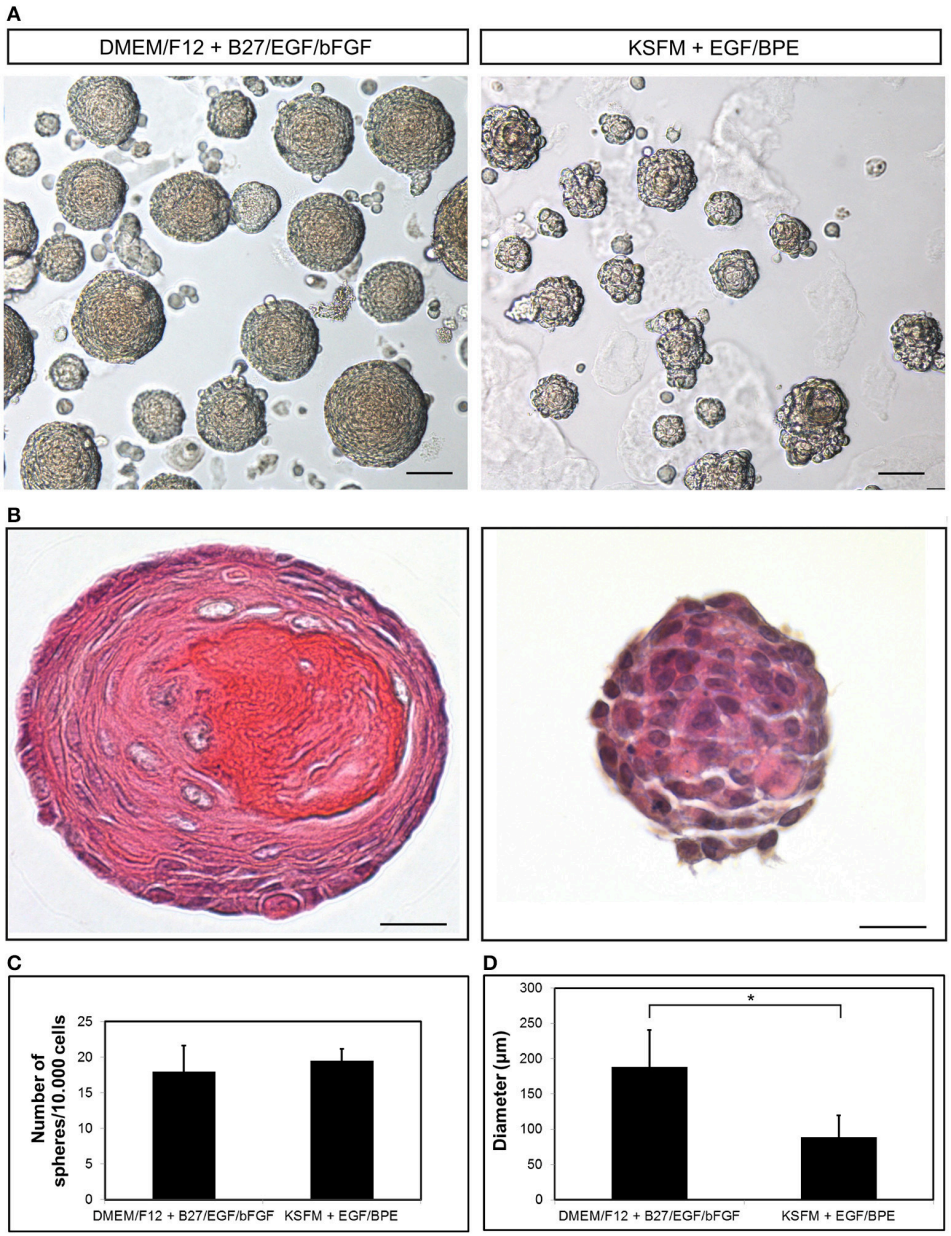
**Growth of dental epithelial stem cells as spheres. (A)** Epithelium-derived dentospheres were formed in presence of two different culture media. **(B)** Hematoxylin-Eosin staining of the resulting spheres. Graphs showing the **(C)** number and **(D)** diameter of the spheres. Scale bars: 50 μm in **(A)**; 20 μm in **(B)**. ^*^p < 0.05.

**Figure 3:**
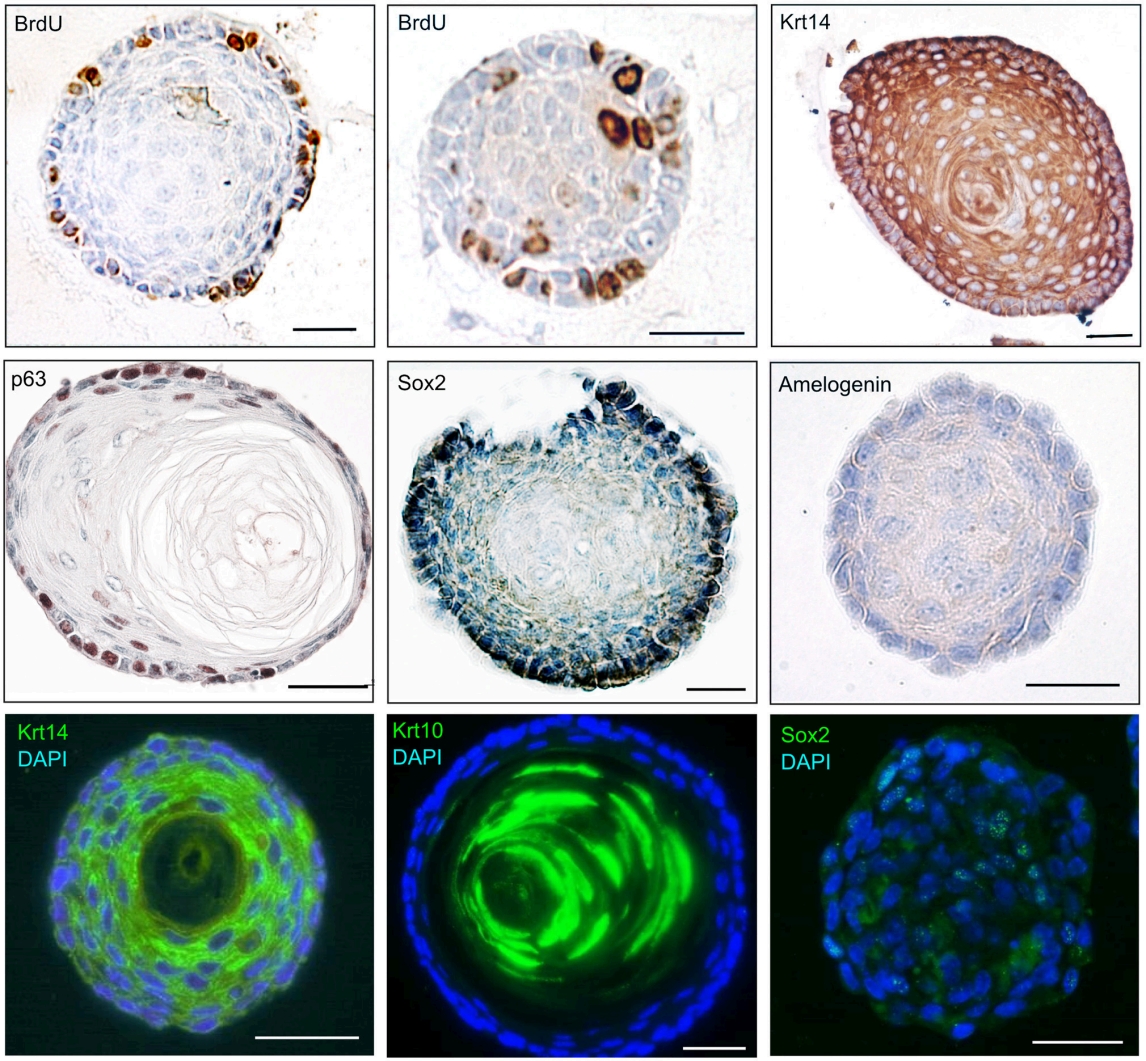
**Molecular profile of epithelial dentospheres**. Immunohistochemistry and immunofluorescence on epithelial dentospheres showing BrdU incorporation in proliferating cells, and expression of specific markers showing the epithelial identity (Krt10, Krt14) and stemness (p63, Sox2) of cells. Absence of Amelogenin indicates that cells within dentospheres are not differentiated into dental specific epithelial cells (ameloblasts). Abbreviations: BrdU, Bromodeoxyuridine; Krt10, Keratin10; Krt14, Keratin14. Scale bars: 30 μm.

We have described the method for the dissection of the cervical loop from mouse mandibular incisors and its further dissociation into a single cell suspension (Figure 1). We have cultured the isolated epithelial stem cells in a three-dimensional (3D) culture system. Epithelial dentospheres have been obtained by incubating dental epithelial stem cells in a 3D culture system using two different culture media (Figure 2A). Thereafter, we have analyzed histologically the dentospheres on 5 μm sections (Figure 2B). Interestingly, although the number of the obtained spheres was not altered when using the two different media (Figure 2C), the morphology and size of the spheres (Figures 2B,D) was affected and was distinct according to the medium used. More precisely, the spheres formed in presence of Dulbecco's Modified Eagle's Medium/Nutrient F-12 Ham (DMEM/F12) medium containing Epidermal Growth Factor (EGF) and basic Fibroblast Growth Factor (bFGF) differentiated into a stratified squamous-like epithelium with a keratinized center (Figure 2A). In contrast, dental epithelial cells cultured in Keratinocyte serum free medium (KSFM) containing EGF and Bovine Pituitary Extract (BPE) formed smaller spheres that lacked any defined organization and not showing a squamous differentiation (Figures 2A,B). Histological sections were used to further analyse the protein expression profile of the spheres by both immunohistochemistry and immunofluorescence (Figure 3). Staining against the dental epithelial stem cell markers Sox2 and p63 indicate the presence of stem cells inside the epithelial dentospheres obtained after culture in presence of both media. In addition, cells within the spheres proliferate, as it is visualized by nuclear Bromodeoxyuridine (BrdU) incorporation, keep their epithelial identity (indicated by Keratin14 expression), and lack differentiated dental cells (indicated by the absence of Amelogenin). Interestingly, the presence of a set of cells expressing the epidermal terminal differentiation marker Keratin10 was observed within spheres cultured in DMEM/F12 implemented with a cocktail of B27/EGF/bFGF molecules (Figure 3). These differentiated cells were less than 15% of the total cell number within the sphere and were situated at the central part of the sphere (Supplementary Figure 1).

## Discussion

Here we describe in detail the various steps leading to the generation of epithelial dentospheres, which were then analyzed histologically and molecularly. Sphere-forming assays were firstly used in the neural stem cell field, where neurospheres were generated from cells of the adult central nervous system (Reynolds and Weiss, 1992). Afterwards, these assays have been largely used to study the behavior and stemness of putative stem cells (Dontu et al., 2003; Yoshida et al., 2005; Edmondson et al., 2014). In teeth, spheres (or dentospheres) have been generated mostly from their mesenchymal component such as the dental pulp and the dental follicle (Miura et al., 2003; Sasaki et al., 2008; Stevens et al., 2008; Abe et al., 2011; Keeve et al., 2013). The formation of spheres originated from dental epithelial tissues retained less attention and therefore remains poorly unexplored. The few realized studies have shown that dental epithelial cells isolated from the cervical loop area of postnatal mouse incisors are able to form spheres in serum-free 3D culture systems (Chang et al., 2013a,b; Chavez et al., 2013, 2014). Interestingly, the resulting spheres presented varying sizes and morphogies depending on the composition of the culture media used in the assays. For instance, when using a medium similar to that used previously for generating neurospheres, which consists of Dulbecco's Modified Eagle's Medium/Nutrient F-12 Ham (DMEM/F12) medium containing EGF and bFGF, dental epithelial stem cells formed well-delimited round-shaped spheres (Chavez et al., 2013, 2014). However, dental epithelial stem cells cultured in an epithelial-specific medium formed spheres that are smaller in size when compared to those obtained with the above mentioned medium (Chang et al., 2013a,b).

Here we show that appropriate selection of signaling molecules is important in order to allow maintenance and proliferation of stem cells within the spheres. Indeed, cells cultured in presence of DMEM/F12 with B27/EGF/bFGF generated spheres with multiple layers, which closely resemble to keratinized stratified squamous epithelial structures. In contrast, in presence of a medium composed by KSFM with EGF/BPE, the spheres adopted a more irregular morphology, where layers were not clearly distiguisable in histological sections. Immunohistochemistry and immunofluorecence revealed that spheres generated in both culture conditions contain cells that proliferate, as shown by BrdU labeling and, furthermore, express the stem cell markers Sox2 and p63. Spheres formed in presence of DMEM/F12 supplemented with B27/EGF/bFGF molecules exhibited a regionalization with clear undifferentiated and differentiated territories, both histologically and molecularly. Indeed, proliferative events and expression of specific stem cell markers were evident in the most external layers of the sphere, thus resembling to the basal layer of keratinized epithelial structures where stem cells reside. In the same spheres, differentiated epithelial cells located in the center could be considered as equivalent to cells of the suprabasal layers of a keratinized epithelium. In contrast, in spheres formed in presence of KSFM with EGF/BPE all cells exhibited a uniform morphology and expressed the Sox2 stem cell marker, but not a terminal differentiation marker, thus suggesting that these spheres contain only cells with stemness.

These results strongly suggest that the epithelial stemness has been retained in the spheres generated by both protocols used. This is reinforced by the capacity of cells originated by the spheres to *de novo* recreate spheres (secondary spheres) when placed in identical culture conditions.

In summary, the methods described here are valuable to assess the stemness of dental epithelial cells, making epithelial dentospheres one promising model for studying tooth pathology and regeneration.

## Author contributions

DN, ZG, and LJ performed the experiments. All authors contributed equally to the experimentation plan, writing and analyzing results.

### Conflict of interest statement

The handling Editor declared a past co-authorship with one of the authors TM and states that the process nevertheless met the standards of a fair and objective review. The other authors declare that the research was conducted in the absence of any commercial or financial relationships that could be construed as a potential conflict of interest.

## Supplementary material

Supplementary Figure 1 **Percentage of undifferentiated vs. differentiated cells within the spheres**. Keratin10 positive terminally differentiated cells constitute less than 15% of the total cell number within the epithelial dentospheres.
