# Supplementary figures and images for: Generation of Spheres from Dental Epithelial Stem Cells
Source: Front Physiol. 2017 Jan 19;8:7. doi: 10.3389/fphys.2017.00007 (PMC5243826; doi:10.3389/fphys.2017.00007)

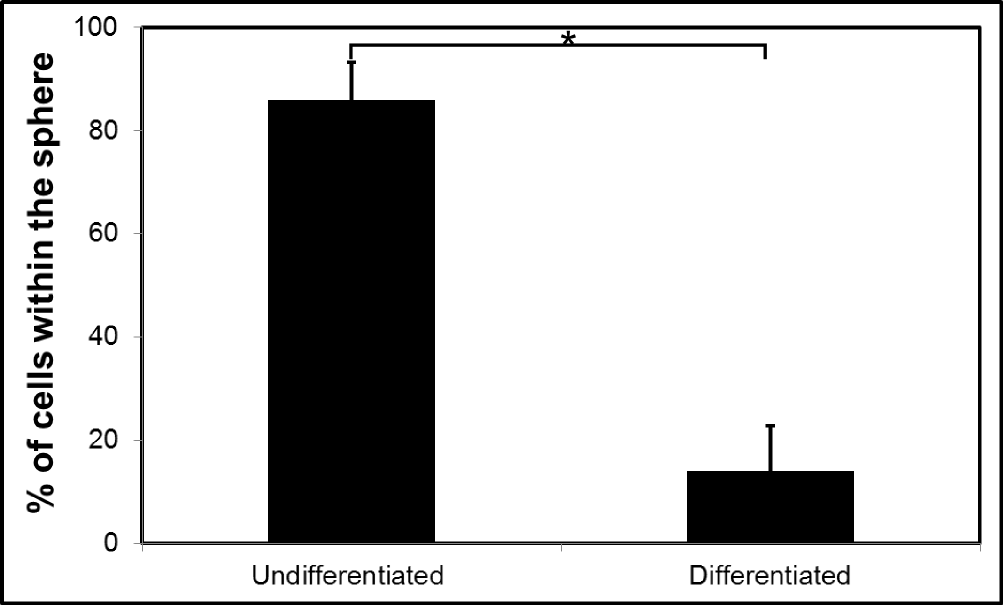

Supplement: Supplementary Figure 1 — Percentage of undifferentiated vs. differentiated cells within the spheres. Keratin10 positive terminally differentiated cells constitute less than 15% of the total cell number within the epithelial dentospheres. [file Image1.TIF]
